# Effect of puberty on the immune system: Relevance to multiple sclerosis

**DOI:** 10.3389/fped.2022.1059083

**Published:** 2022-12-02

**Authors:** Carmen C. Ucciferri, Shannon E. Dunn

**Affiliations:** ^1^ Department of Immunology, The University of Toronto, Toronto, ON, Canada; ^2^ Keenan Research Centre for Biomedical Science of St. Michael’s Hospital, Toronto, ON, Canada; ^3^ Women's College Research Institute, Women's College Hospital, Toronto, ON, Canada

**Keywords:** puberty, autoimmunity, T helper cells (Th cells), antigen presenting cell (APC), gonadal hormone, multiple sclerosis, experimental autoimmune encephalitis (EAE)

## Abstract

Puberty is a dynamic period marked by changing levels of sex hormones, the development of secondary sexual characteristics and reproductive maturity. This period has profound effects on various organ systems, including the immune system. The critical changes that occur in the immune system during pubertal onset have been shown to have implications for autoimmune conditions, including Multiple Sclerosis (MS). MS is rare prior to puberty but can manifest in children after puberty. This disease also has a clear female preponderance that only arises following pubertal onset, highlighting a potential role for sex hormones in autoimmunity. Early onset of puberty has also been shown to be a risk factor for MS. The purpose of this review is to overview the evidence that puberty regulates MS susceptibility and disease activity. Given that there is a paucity of studies that directly evaluate the effects of puberty on the immune system, we also discuss how the immune system is different in children and mice of pre- vs. post-pubertal ages and describe how gonadal hormones may regulate these immune mechanisms. We present evidence that puberty enhances the expression of co-stimulatory molecules and cytokine production by type 2 dendritic cells (DC2s) and plasmacytoid dendritic cells (pDCs), increases T helper 1 (Th1), Th17, and T follicular helper immunity, and promotes immunoglobulin (Ig)G antibody production. Overall, this review highlights how the immune system undergoes a functional maturation during puberty, which has the potential to explain the higher prevalence of MS and other autoimmune diseases seen in adolescence.

## Introduction

Puberty is a physiological stage of life when a child becomes capable of sexual reproduction (1). Puberty is initiated by an increase in the pulsatile release of gonadotropin releasing hormone (GnRH) from neurons in the hypothalamus, which triggers gonadotropin secretion from the pituitary and the subsequent maturation of the gonads (1). Outwardly, puberty manifests as the development of secondary sex characteristics including pubarche (growth of pubic and axillary hair), voice lowering and facial hair growth in males, and breast development and hip widening in females. However, the rise in gonadal hormones initiates physiological changes in other organ systems, including the immune system (1). In this regard, puberty serves as a point of inflection for increased development of T cell-mediated autoimmune diseases, which includes multiple sclerosis (MS) (2, 3).

MS is an autoimmune disease that targets central nervous system (CNS) myelin (4). MS is rare prior to puberty and the incidence of this disease increases drastically in children of post-pubertal ages, particularly in females (5, 6). There is emerging evidence that the environmental factors that contribute to MS, including the development of obesity, repeated concussion injury, and Epstein Barr infection regulate MS susceptibility in adolescence as opposed to childhood or adulthood. This further points to puberty as a time when the immune system is more prone to autoimmunity development (7). Experiencing early puberty is also a risk factor for the development of MS in women (8). How puberty modifies the immune system to stimulate autoimmune mechanisms in MS is not completely understood. Here, we overview the relationship between puberty, pubertal timing and MS risk. We also discuss how puberty enhances the development of CNS autoimmunity in MS and in the animal model of MS, experimental autoimmune encephalitis (EAE). Finally, we review how the immune landscape changes from pre- to post-pubertal ages to better understand how puberty enhances cellular and humoral immune mechanisms that are relevant to MS.

## The hypothalamus-pituitary-gonadal axis and hormonal changes with puberty

The hypothalamus-pituitary-gonadal (HPG) axis is the physiological cascade that triggers the onset of puberty and gonadal hormone production (9). The HPG axis is first activated prenatally in humans, becomes quiescent shortly after birth, and remains suppressed during childhood before resuming activity just prior to puberty (9). The onset of puberty is characterized by an increase in the pulsatile release of GnRH from neurons in the hypothalamus (9). GnRH is released into the portal circulation where it binds to GnRH receptors on gonadotropin cells in the anterior pituitary (9). GnRH signaling stimulates the secretion of luteinizing hormone (LH) and follicle-stimulating hormone (FSH) from the pituitary, which stimulate gonadal maturation and the synthesis of gonadal hormones (9). Gonadal hormones also feedback on the HPG to regulate the release of GnRH; this negative feedback regulation forms the basis of the menstrual cycle in females (9). The maturing gonads secrete gonadal hormones including testosterone, estradiol (E2) and progesterone and these hormone levels gradually rise throughout puberty (9). All three of these hormones are present in males and females, however, there is a sex disparity in their levels, with adult males exhibiting higher levels of testosterone than females and females exhibiting higher levels of E2 and progesterone than males (10). It is the sex disparity in testosterone and E2 levels that is considered to be the major factor regulating sex differences in the immune system (11).

It is still unclear what initiates the pulsatile release of GnRH at the onset of puberty. It is thought that GnRH^+^ neurons undergo synaptic pruning and dendrite reorganization during puberty to help regulate inputs provided by both inhibitory (GABA-producing) and excitatory (glutamate- and Kisspeptin-producing) neurons (12). Kisspeptin is a neuropeptide that is crucial for the initiation of puberty and the expression increases in a population of neurons in the hypothalamus that also express neurokinin B and dynorphin (called KNDy neurons) just before pubertal onset (13). Kisspeptin is released from the nerve terminals of KNDy neurons and binds to its receptors on GnRH^+^ neurons to activate GnRH secretion [12]. What triggers the initial expression of *Kiss1* in KNDy neurons just prior to puberty is also not well understood. One hypothesis is puberty occurs because of a decreased sensitivity of *Kiss1* to negative regulation by gonadal hormones. KNDy neurons express both the estrogen receptor (ER)-α and the androgen receptor (AR) (13). Providing rodents an oophorectomy surgery prior to puberty increases GnRH secretion, even in pre-pubertal animals, indicating that the low levels of E2 present prior to puberty are sufficient to suppress the HPG axis (14). Furthermore, deletion of *Esr1* (that encodes ER-α) from glutaminergic neurons, hastens puberty (15). Puberty is also accelerated by metabolic signals including leptin levels that rise in response to the increase in adiposity. Leptin positively regulates *Kiss1* expression in KNDy neurons indirectly to regulate gonadotropin secretion in the pituitary (16). Overall, there is a very complex interplay between estrogen and metabolic inputs on GnRH^+^ and KNDy neurons during the pubertal period that regulates the onset of puberty, sexual maturation, and the increased production of gonadal hormones.

## Effects of puberty and pubertal timing on MS incidence and disease activity

### The prevalence of MS before and after puberty

The effect of puberty on MS can be appreciated by comparing the incidence of MS in male and female children of different ages or pubertal statuses. A large study of pediatric MS patients in the United States observed that a large majority (85%–89%) of pediatric MS cases have a post-pubertal onset (5, 6, 17). In this study, pubertal status was estimated by Tanner staging and by age at menarche, which is the age at which females experience first menses. Similar observations were seen in German (18) and a multi-national (6) studies of pediatric MS that examined the prevalence of MS in children of pre- and post-pubertal ages. A common observation in these studies is that the female to male ratio of MS emerges post-puberty, being 1 : 1 in children of pre-pubertal ages and 2–3 : 1 in children of post-pubertal ages (5, 6, 18, 19). One of these studies further reported that the peak age of onset of pediatric MS trailed menarche by 2 years (4), suggesting that changes in the immune system triggered by puberty can take years to manifest into the first MS clinical attack in humans. Together, these data suggest that puberty is a point of inflection of increased MS risk, particularly in female children.

### Effect of pubertal timing on MS risk and progression: relationship to childhood obesity

A number of studies have observed a relationship between early menarche in females and increased MS risk. Particularly, each one-year increase in menarche is associated with an approximately 10% decrease in the risk of MS (5, 8, 17, 21–29) (summarized in Table 1). This effect of pubertal timing on MS risk is not apparent in males (5, 8, 17). However, it is controversial whether the effect of early menarche on MS risk relates to pubertal timing or increased adiposity. Some studies that explored the relationship between age at menarche and MS risk also measured body mass index (BMI) or estimated body size at the time of puberty (5, 17, 27, 29) and found that increased BMI had a comparatively stronger effect than early menarche in predicting MS (5, 17, 29). However, since the age of puberty and adiposity are inextricably linked, it has been difficult to distinguish which of these two factors is the true enhancer of MS disease mechanisms. Recently, Mendelian randomization studies have been performed to sort out the role of these two factors in MS. These studies examined the effects of genetic polymorphisms that associate with early menarche or childhood obesity on MS development (17, 30). These studies concluded that genetically predicted age at puberty and BMI both associate with increased MS risk but when BMI is controlled for, the association between early menarche and MS disappears (17, 30). These results suggest that it is the increase of fat mass in girls who experienced early menarche that is the major driver of the increased MS risk. Indeed, there is evidence that increased adiposity can enhance the severity of the animal model of MS, called experimental autoimmune encephalomyelitis (EAE) (31–35). Short-term (2–10 weeks) diet-induced obesity (DIO) enhances T helper cell-mediated inflammation in the CNS in EAE (31). This intervention also increases IL-6 and Th17 cytokine production in male mice (32, 33). This effect of DIO in enhancing Th17 cell response may relate to the upregulation of acetyl-CoA carboxylase 1 in the T cells, which is a regulator of Th17 cell differentiation (33). These studies in mice thus provide a potential mechanism for how increased adiposity in adolescence may accelerate the development of CNS autoimmunity.

**Table 1:** Summary table of studies that examined the relationship between early menarche and MS risk. Studies including adult MS cases were limited to those who sampled age of puberty in at least 100 MS cases and controls. Pediatric studies that had smaller samples are included.

Study	Region	Type of study	Populations studied (*N*)	Puberty measurement	Findings
Operskalski et al. 1989 (21)	US	Case control Adult MS	MS Cases (145) Friend controls (145)	Age at menarche	– Mean age of menarche in MS cases less than controls.
Ramagopalan et al. 2009 (8)	Canada	Case Control Adult MS	MS Cases (4472 F, 1,021 M) Spousal Controls (658 F, 1,101 M)	Information about at menarche, age of voice change, and pubarche collected by interview	– Mean age of menarche lower in female MS cases (12.4) compared to female controls (12.6) – Age at puberty not different between male MS cases and controls – There is a 10% reduction in the relative risk of MS for each year increase in age at puberty
Gustavsen et al. 2014 (22)	Norway	Case control Adult MS	MS cases from Oslo MS Registry (391 F, 139 M) Controls from Norwegian Bone marrow registry (535 F, 383 M)	Age at menarche information collected by questionnaire	– Mean age at menarche was not different in MS patients (13.1) compared to controls (13.0)
Chitnis et al. 2016 (5)	US	Case Control Pediatric MS	MS Cases (160 F, 94 M) Age, sex, and race-matched controls from same hospitals (206 F, 214 M)	Age at menarche, BMI, and pubertal staging data on both males and females collected at time of study visit.	– Age of menarche earlier in MS patients (11.6) compared to controls (11.9). – BMI higher in MS cases (76) vs. controls (63) – Among females, there is a correlation between higher BMI and younger age at menarche (this was not apparent not in boys) – There was an effect of BMI on MS risk only in post- but not pre-menarche girls (OR = 1.60). – Age of onset of symptoms occurred 0.91 years earlier for overweight females, particularly if they experienced puberty earlier.
Bove et al., 2016 (23)	US	Case control Adult MS	MS or CIS cases were white females from the CLIMB study (156) and 1,390 white women without MS but with a first-degree relative with MS from [Genes and Environment in Multiple Sclerosis (GEMS)].	Age at menarche and information about obesity in childhood (yes/no) collected by questionnaire	– Age at menarche similar between index cases (12.6) and controls (12.7). – Every year decrease in age at menarche was associated with a 0.65-year earlier MS onset
Rejali et al. 2016 (24)	Iran	Case control One Hospital Adult MS	MS Cases (200 F) Age-matched controls (200 F)	Assessment of age of onset of puberty was made by Tanner classification and age at menarche	– Age at menarche earlier in MS cases (13.0) compared to controls (13.5). – Inverse relationship between age of menarche and MS risk (OR = 0.78)
Nielsen et al. 2017 (26)	Denmark	Prospective Cohort Adult MS	Women enrolled in the Danish National Birth Cohort MS Cases (226 F) Controls (77 330 F)	Information about age at menarche was collected by interview at week 16 of pregnancy	– Mean age at menarche was earlier in female MS cases (13.0) compared to controls (13.3). – There was a 13% reduction in the relative risk of MS for each year increase in age at menarche. – Early menarche (<11 yo) is associated with 80% increased risk of MS, compared to late menarche (>15) (HR = 1.8). – Comparable hazard ratios seen after adjusting for co-variates

Salehi et al. 2018 (25)	Iran	Case control Adult MS	MS Cases (399 F) registered with Iranian MS society Controls (541 F)	Information about age at menarche collected by questionnaire	– Mean age of menarche in MS cases (13.14) was earlier than controls (13.36). – 1-year increase in age at menarche reduced MS risk by 10% (OR = 0.90)
Mirmosayyeb et al. 2018 (27)	Iran	Case control One hospital Adult MS	MS Cases (181 F, aged >14 years) Age-matched controls (202 F)	Information about age at menarche collected by interview.	– Mean age at menarche was not significantly different between MS cases (13.6) and controls (13.3) – MS cases did not exhibit higher body mass index than controls.
Belbasis et al., 2020 (17)	UK	Case Control Adult MS	MS Cases (1,381 F) Controls (229,197 F) UK Biobank	Age at menarche and body size at age 10 information collected at time of sample collection in Biobank	– Age at menarche earlier in MS cases (12.77) compared to controls (12.95). – One age later in menarche associated with protection from MS (OR = 0.93). – Having a higher-than-average body size at aged 10 also associated with higher risk of MS. – No significant association with age at which voice broke and age of first facial hair with risk of MS.
Zeydan et al. 2020 (28)	US	Case control Adult MS	MS Cases (137 F) Controls (396 F) were from the Mayo Clinical Cohort Study of Oophorectomy and Aging, and were matched with MS cases for year of birth.	Information about age at menarche collected by questionnaire and interview	– Mean age at menarche not different between MS cases (12.7) compared to controls (12.6).
Jacobs et al. 2021 (29)	UK	Case control Adult MS	MS Cases (1,635 F) Controls (263,058 F) UK Biobank	Information about age of menarche and body size at age 10 information collected from UK Biobank database.	– Earlier age at menarche and higher BMI associated with MS risk.

There is evidence that early menarche or accompanying increases in adiposity also enhance MS disease activity in children. In a Canadian prospective study that examined disease outcomes in female children who experienced an incident demyelinating event, it was found that each year increase in age at menarche associated with a 36% decrease in the probability of having a second demyelinating event and a diagnosis of MS (36). Children who were more susceptible to having a second attack also had higher BMI percentiles than the children who did not (36). By contrast, a large study of adult women who experienced an incident demyelinating event (termed Clinical Isolated Syndrome or CIS) reported that age-at-menarche had no predictive value for MS diagnosis (37); However, the difference in this study is that BMI at menarche was adjusted for, thereby removing the influence of BMI on disease activity. These findings reinforce the idea that increased adiposity may underlie the effect of pubertal timing on MS.

Studies by Waubant and colleagues who followed children with MS prospectively were able to directly study the effect of puberty on MS disease activity. They compared annualized relapse rates in their patients in the pre-, post-, or peri-pubertal periods, which were defined as the 6 months before, after, or spanning menarche onset in females. In the boys, these pubertal stages were estimated by landmark ages (20, 38). Though the number of children sampled was small, relapse rates were found to be significantly higher in the peri-pubertal period in both boys and girls (20, 38). Furthermore, BMI increased from the pre to the peri-pubertal period, but was not different between the peri- and post-pubertal periods (20). These findings suggest that the hormonal environment associated with puberty is driving enhanced autoimmune mechanisms. If autoimmunity is indeed initiated at the time of puberty, it would go further in explaining the positive correlation between age at menarche and age of MS onset observed in some studies (5, 23).

A few studies have also investigated the relationship between menarche onset and disability progression in MS. D'Hooghe and colleagues reported that while age at menarche did not alter the probability of MS patients reaching an expanded disability status scale (EDSS) of 6 (a disability landmark when patients need a cane to ambulate), those with progressive-onset MS who experienced menarche after aged 13 years were 40% less likely to reach this disability landmark; BMI was not considered in this study (39). By contrast, in the study of CIS that adjusted for BMI, it was reported that age-at-menarche had no predictive value on time to reaching EDSS3 or EDSS6 (37). The underlying mechanisms for how a later age at menarche may slow the onset of progressive MS and its relationship to past or current BMI remain unknown.

In conclusion, these findings suggest that the peri-pubertal hormone environment is more conducive to the initiation of MS, particularly in females, and that increased adiposity may be a factor in the effects of pubertal timing on MS disease activity.

## Artificial enhancement of the HPG axis triggers relapses in MS

Assisted reproductive techniques (ART) are used to overcome infertility in women (40). Usual procedures require suppression of the HPG axis, followed by hyper-stimulation of ovaries with gonadotropins to induce ovulation in a controlled manner. Progesterone support is also given during the luteal phase of the cycle, prior to fertilization (41). Suppression of the HPG axis is achieved by treatment with either agonists or antagonists for the GnRH receptor, which both have the same effect of suppressing GnRH receptor signaling. The major difference between these modes of HPG suppression is that GnRH agonists initially cause the secretion of gonadotropins and gonadal hormones prior to downregulation of GnRH receptor expression (42).

It has been reported that up to 14% of women diagnosed with MS undergo ART treatment (43). A number of studies have investigated the effects of ART on MS disease activity (44–48). While these investigations were individually limited by small sample sizes, a meta-analysis of combined data concluded that ART increases relapse rates in the 3-month period following treatment (44). Interestingly, ART protocols that use GnRH receptor agonists and antagonists both increase relapse rates, suggesting a role for ovarian stimulation in this effect. Nonetheless, the protocols that used GnRH receptor agonists had an even more profound effect in increasing relapse rates (44). A Brazilian study reporting on ART using GnRH receptor agonists, found that ART induced a 7-fold increase in the relapse rate in the MS patients compared to baseline levels (45). This ART regimen induced a marked increase in the levels of ovarian hormones and an increase in the expression of the GnRH receptors on immune cells at 10–14 days post-treatment. Furthermore, when myelin-specific T cells were grown from the blood of MS patients collected before and after ART, it was found that cells isolated post-ART exhibited much higher proliferation and Th1 cytokine production following *in vitro* anti-CD3 stimulation. These results suggested that an increased number of pro-inflammatory T cells were present in the blood at 3 months post-ART (45). Post-ART MS patients also exhibited a higher frequency of MOG-specific IgG producing cells in the blood as detected by ELISPOT (45). Interestingly, GnRH was also found to be produced at higher levels by immune cells post-ART and this peptide had an effect of enhancing myelin-specific T cell responses and MOG-specific antibody production *in vitro*. These findings of ART in MS are striking and provide proof of concept that stimulation by GnRH enhances autoimmune mechanisms in MS. These findings of GnRH agonists on the immune system further raise the possibility that GnRH or gonadotropins, may be additional players in the enhanced CNS autoimmunity seen post-puberty.

## Puberty alters the epigenetic landscape of human immune cells

In an effort to understand how the immune system changes with puberty, Thompson and colleagues studied whole genome DNA methylation patterns in peripheral blood mononuclear cells (PBMCs) of males and females before and after puberty (49). They found 445 differentially methylated regions (DMRs) in the PBMCs of pre- and post-pubertal children with the majority (78%) being unique to girls, 11.2% being unique to boys and the remaining shared between the sexes. Network analysis on the genes near the DMRs identified pathways involved in both immune and reproductive function. Furthermore, the majority of genes located near the DMRs in females contained estrogen response elements (49), consistent with the idea that E2 was mediating some of these changes in methylation.

Similarly, a longitudinal study in Denmark that explored genome-wide DNA methylation patterns in 22 girls and 32 boys before and after puberty identified several CpG islands that closely tracked with pubertal transition in boys; none significantly tracked with puberty in girls (50). It was speculated that the lack of association in girls was related to the analysis being underpowered to detect differences. The most significant region for both sexes, when analyzed in boys and girls alone or together, was a region on chromosome 7 that contained the last exon and 3′ UTR of *SLC12A9* and the promoter of *TRIP6*. Changes in methylation levels in this genomic region significantly associated with testosterone levels in boys and FSH and LH levels in both sexes. While the function of *SLC12A9* has not been studied in the immune system, the function of *TRIP6* has. This protein can associate with TRAF6 to induce its oligomerization and polyubiquitination, which triggers the activation of the Nuclear factor kappa-light-chain-enhancer of activated B cells (NF-κB) signaling pathway (51). There is also evidence that the presence of TRAF6 can promote inflammation in murine models of colitis (51). Thus, the upregulation of *TRIP6* could be one means by which the human immune system matures with puberty. Taken together, these studies suggest that puberty affects the epigenetic landscape of human immune cells; however, more studies are needed to understand how the immune system is functionally different before and after puberty.

## Puberty alters autoimmune mechanisms in MS animal models

It is difficult to study how autoimmune mechanisms change with puberty to initiate MS, since blood samples are typically collected after puberty and after the disease has already started. Studies in animal models help to fill this knowledge gap. EAE is a murine model for CNS autoimmunity that shares many pathological features with MS including the CNS infiltration of granulocyte-macrophage colony-stimulating factor (GM-CSF)-producing Th1 and Th17 cells, inflammatory monocytes, demyelination, and axon injury (52–58). In contrast to the situation in MS where autoantigen is unknown, the autoantigen in EAE is controlled and is used to induce the disease. EAE is most commonly induced in susceptible strains of rodents by immunization with an emulsion containing myelin proteins or peptides and Complete Freund's Adjuvant (CFA), sometimes with co-administration of pertussis toxin (59). EAE can also be induced by transfer of myelin-specific T cells into naïve recipient mice: This mode of induction is called passive EAE or adoptive transfer EAE (60). In adoptive transfer EAE, draining lymph nodes and spleens containing myelin-reactive T cells are collected from mice pre-immunized with myelin antigens and CFA. These cells are reactivated *ex vivo* with antigen, and then transferred into naïve recipient mice. In addition, EAE can also occur spontaneously in mice that overexpress T cell receptors (TCRs) specific for myelin antigens (61).^.^

In the naïve state in both humans and mice, myelin-specific T helper cells develop in the thymus and seed the peripheral lymphoid organs; however, these T cells remain in a state of ignorance unless they encounter self-antigen major histocompatibility complex II (MHC II) (signal 1) in the context of co-stimulatory receptors (B7.1/B7.2) (signal 2) on the same DCs. In EAE, danger signals provided by the CFA activate toll-like receptors on DCs that trigger the upregulation of co-stimulatory molecules and production of IL-6 and IL-12 family cytokines by these cells (signal 3) (62). These changes license the DCs to activate myelin-specific T helper cells, which then proliferate and differentiate into Th1 and Th17 cells in the draining lymph nodes and spleen. These Th cells then circulate through the blood and lung before entering the CNS through blood-meningeal and blood brain barriers (63). At CNS barrier sites, myelin specific Th1 and Th17 effector cells encounter myelin peptides presented by local antigen presenting cells (APCs). These autoreactive T cells become re-activated and produce chemokines to recruit other leukocytes to the CNS, as well as effector cytokines, including interferon-γ (IFN-γ), interleukin (IL)-17, and GM-CSF, which can promote inflammation and lesion formation in the CNS white matter.

A number of studies have evaluated the effect of puberty on EAE (Section “Preventing puberty in female SJL mice increases resistance to EAE by keeping DCs in an immature state”) or have compared the development of EAE in mice of pre- and post-pubertal ages (Sections “Neonatal mice are resistant to EAE compared to adults” and “Mice that are transgenic for myelin specific TCRs are vulnerable to EAE in the weeks immediately following onset of puberty”), thereby providing insights into how puberty may enhance autoimmune mechanisms.

### Preventing puberty in female SJL mice increases resistance to EAE by keeping DCs in an immature state

**Figure 1:**
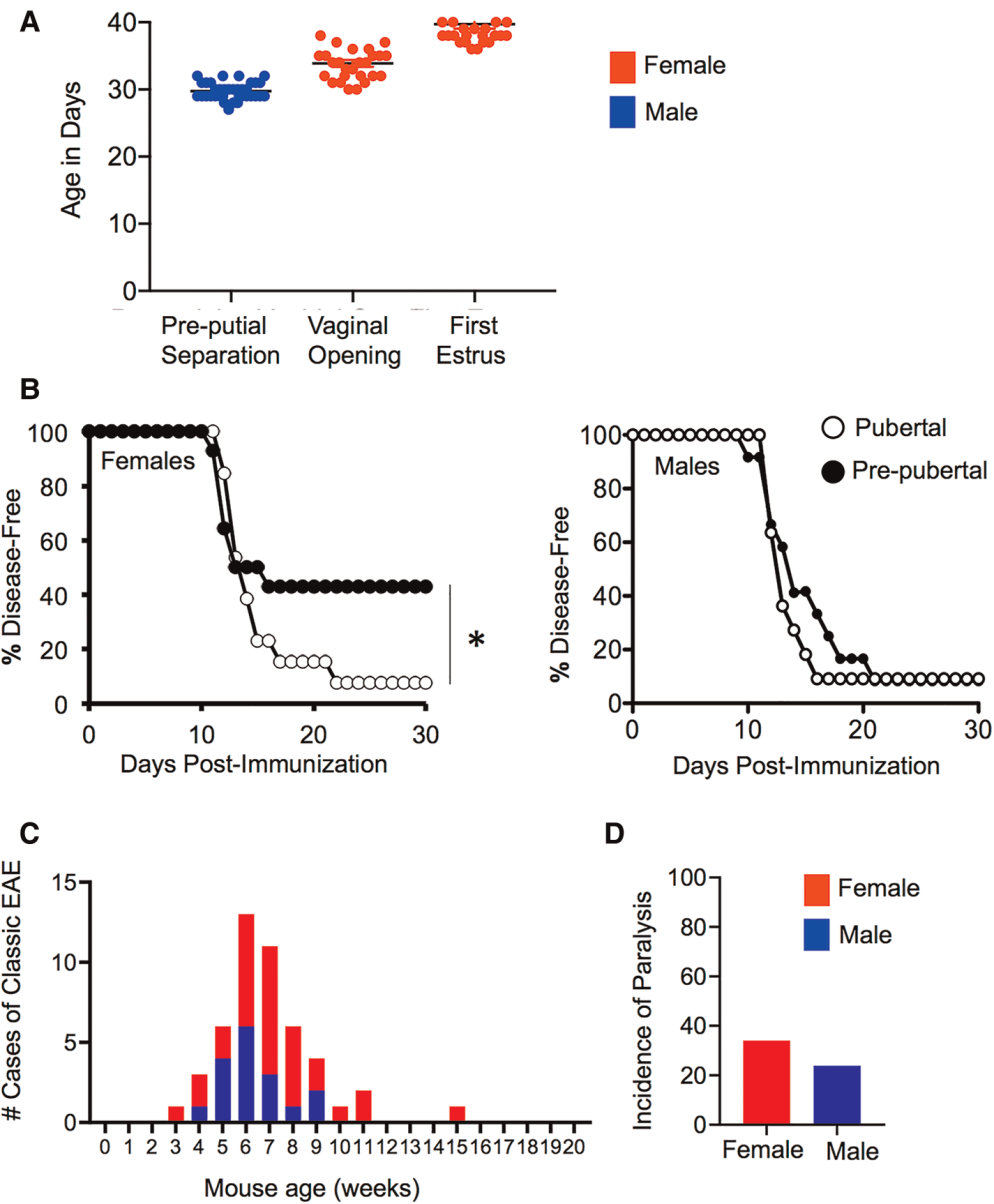
Mice are more prone to EAE post-puberty in induced and T cell receptor transgenic models. (**A**) Puberty can be estimated in female mice by measuring vaginal opening (first sign), which is confirmed by first estrus or in male mice by measuring the age at pre-putial separation (when the prepuce can be easily separated from the glans penis). Shown is the mean + SEM age of pre-putial separation in male (*N* = 36), and vaginal opening and first estrus in female (*N* = 28) C57BL6/J mice. (**B**) Incidence of EAE observed in female and male SJL/J mice induced at 8 weeks of age with PLP p139-151/CFA that had received a pre-pubertal gonadectomy or sham surgery at the age of 4 weeks. Data shown represents the disease-free survival in mice over 30 days of observation. *N* = 14 for pre-pubertal females (pre-pubertal oophorectomy), *N* = 15 for post-pubertal female (pre-pubertal sham surgery), *N* = 11 for pre-pubertal male (pre-pubertal castration), *N* = 12 for post-pubertal male (pre-pubertal sham surgery). *Significant by Log-rank test (*P* < 0.05). (**C,D**) The number of cases of spontaneous EAE (**C**), and the incidence of paralysis (**D**) in MOG TCR transgenic mice over a period of 2 years.

In a mouse, pubertal onset occurs between the age of 28 and 35 days as estimated by examining the age at vaginal opening in females (which is confirmed by first estrus) (64) or by determining the age of pre-putial separation in males (65) (Figure 1A). Accordingly, surgical removal of the gonads in mice between 3 and 4 weeks of age can suspend development in the pre-pubertal state. The effects of puberty can be studied by comparing the phenotype of pre-pubertally gonadectomized adult mice and age-matched mice that receive a sham surgery and proceed through puberty normally.

Using this approach, Ahn and colleagues (36) contrasted the effect of pre-pubertal gonadectomy or sham surgery on the development of proteolipid protein peptide (PLP p139–151)/CFA-induced EAE in eight-week-old SJL/J mice. They observed that post-pubertal female mice exhibited an increased incidence of both active and adoptive transfer EAE compared to age-matched pre-pubertal counterparts that had been oophorectomized prior to puberty. However, EAE incidence did not vary between the pre- and post-pubertal age-matched males (see Figures 1B,C). Further studies using the active EAE model in female mice demonstrated that post-pubertal animals exhibited a comparatively larger expansion of myelin-specific T cells in peripheral lymphoid organs than that seen in pre-pubertal mice. This was accompanied by enhanced production of PLP-specific IgG, but not IgM or IgA in the post-pubertal mice. This phenotype did not associate with an altered Th1/Th17/Th2 cytokine balance, differences in the intrinsic activation potential of CD4^+^ T cells, or alterations in the frequency or function of Foxp3^+^ T regulatory cells (Treg) (36). Instead, the enhanced EAE incidence seen post-puberty correlated with increased maturation of APC (36). Post-pubertal splenic CD11c^+^ cells had a higher expression of co-stimulatory molecules CD80, CD86, and CD40 in the steady state and produced higher IL-12p40 compared to pre-pubertal counterparts. Post-pubertal B cells also expressed higher levels of MHC II. Both of these APC populations were more efficient at priming PLP p139–151-reactive T cell receptor transgenic CD4^+^ T cells *in vitro* in the presence of added PLP peptide or whole PLP antigen. Importantly, in this model, the pubertal mice did not exhibit altered fat masses or body weights compared to pre-pubertal mice, suggesting that the differences in immune phenotype between pre- and post-pubertal females was due to differences in the sex hormone environment rather than differences in adiposity.

Coinciding with these findings in EAE, pre-pubertal oophorectomy also protects against the development of type I diabetes (66, 67) and systemic lupus erythematosus (68) in murine models. The protective effect of oophorectomy against development of autoimmunity in these models was achieved only when the surgery was done prior to puberty, but not at seven-to-eight weeks of age (67, 68), suggesting that female gonadal hormones somehow imprint the immune system to be more prone to autoimmunity in the period post-puberty. In the murine lupus study, protection afforded by pre-pubertal oophorectomy coincided with a striking paucity of myeloid CD11c^+^CD11b^+^ cells (also known as type 2 dendritic cells or DC2 cells) in the spleen (68). DC2 cells are a DC subtype that is most efficient at presenting antigen *via* MHC Class II to T helper cells (69). Similar to these findings in mice, neonatal humans are reported to have reduced numbers of CD11b^+^CD11c^+^ in the circulation compared to that in adults (70). Human DCs from neonates are also reported to have a more immature phenotype compared to adult DCs with lowered expression of co-stimulatory molecules, a lower potential to produce pro-inflammatory cytokines (TNF, IL-6, IL-12p40, IL-1), and a reduced capacity to prime T helper cells [reviewed in Ref. (71)]. Thus, the maturation of the DC compartment with age and/or puberty is a conserved feature between humans and mice.

**Figure 2:**
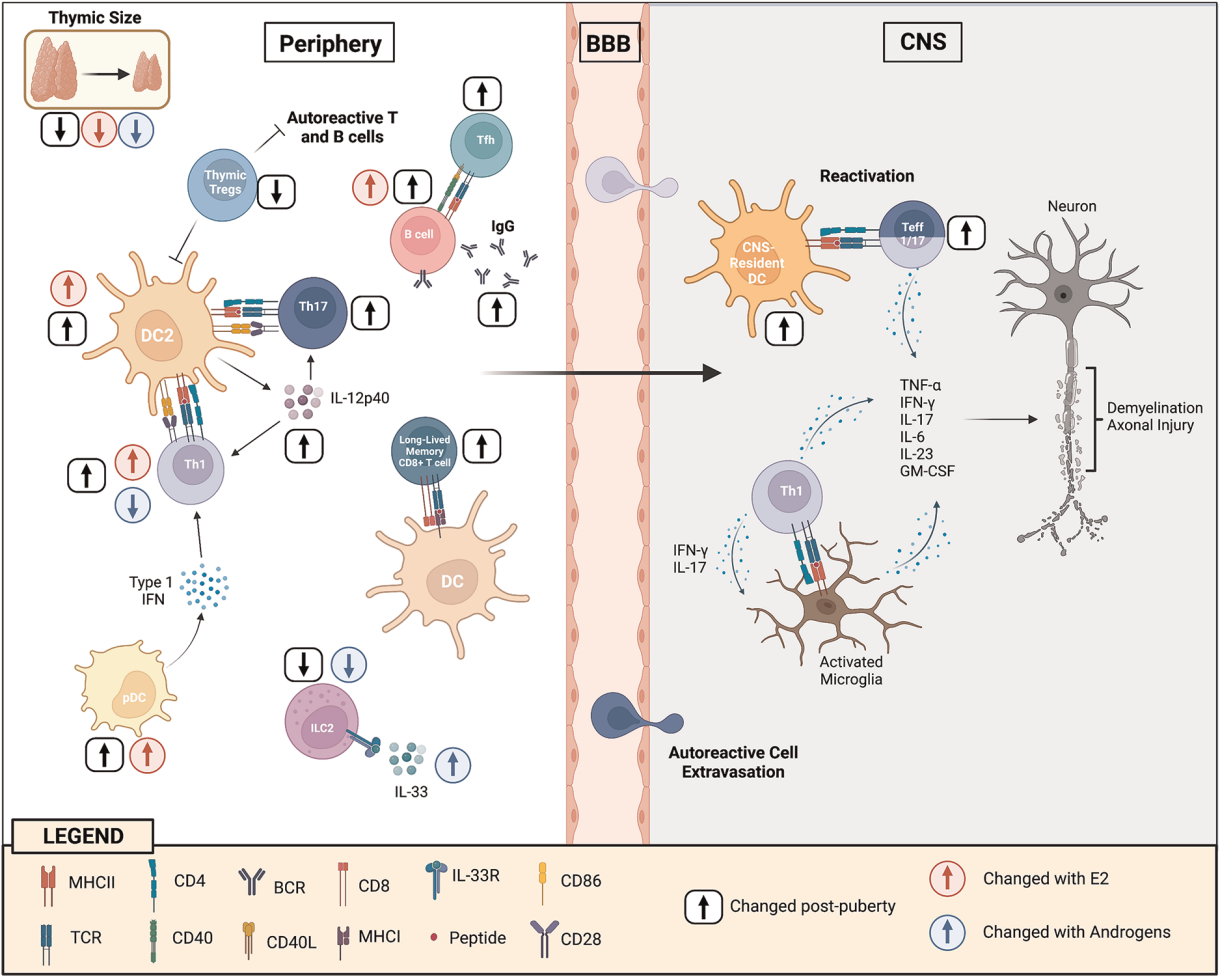
Puberty augments cellular and humoral immunity to enhance CNS autoimmunity. During puberty, DC2 cells become more mature and efficient at presenting antigen to T helper cells resulting in enhanced Th1 and Th17 immunity. This occurs, in part, through increases in the expression of co-stimulatory molecules, but also IL-12p40 (and IL-12 and IL-23) production by these cells. Adoptive transfer EAE studies in mice of pre- and post-pubertal ages have provided evidence that post-pubertal DCs in the CNS may have a higher potential to re-activate Th1/Th17 cells that have reached the CNS site, resulting in increased myelin tissue damage. Increases in E2 with puberty induce higher levels of type I interferon by pDCs which may further enhance Th1 responses. There is evidence that Th1 cells develop more in a female during the post-pubertal state because of the promoting effects of E2 and the inhibiting effects of androgens. T follicular cell responses also develop more efficiently post-puberty which helps promote B cell responses in the germinal center, resulting in higher IgG production. E2 has an effect in increasing the threshold for BCR activation, allowing potential autoreactive B cells to escape deletion in the periphery. The thymus undergoes dramatic changes with puberty resulting in reduced generation of naïve T cells, in particularly Tregs. This loss in thymic output of T cells may trigger the homeostatic proliferation of perinatal T cells that have a higher TCR responsiveness to self-antigen. With maturation, the BCR and TCR responsiveness to self-antigens becomes diminished, favoring the formation of long-lived memory B cell and CD8^+^ T cell memory cells instead of short-lived effectors. In addition, ILC2 cell numbers decline post-puberty, in part due to suppressive effects of androgens in the bone marrow. Post-pubertal male ILC2s are also more responsive to IL-33 stimulation. Androgens induce ILC2 cells to accumulate in the lymph nodes of male mice during EAE, limiting disease development in this sex through promotion of the Th2 response.

DCs are short-lived cells that are continuously being generated from progenitors in the bone marrow. There is strong evidence that the increase in CD11b^+^CD11c^+^ DCs seen with puberty in females is, in part, due to E2 effects on the bone marrow. When bone marrow pre-cursors are cultured in the presence of GM-CSF, treatment with E2 (and ER-α agonists, but not AR agonists) enhances the yield of CD11b^+^CD11c^+^ DCs [reviewed in Ref. (72)]. ER-α signaling appears to promote CD11b^+^ DC differentiation by increasing the expression of interferon regulatory factor 4 in DC precursors, which is a transcription factor that is crucial to myeloid DC development (73, 74). The importance of E2 in DC differentiation is exemplified by the findings that when mice were irradiated and re-constituted with a mix of ERα^+/+^ and ERα^−/−^ bone marrow (75), newly differentiated CD11b^+^ DCs derived primarily from the ERα^+/+^ pre-cursors (75). DCs that are generated from bone-marrow precursors in the presence of E2 have been found to be also functionally more competent at priming T helper cells, which correlates with higher expression of MHC II and co-stimulatory molecules, and an enhanced potential of these cells to make IL-12p40 and other cytokines (76–78). Therefore, there is strong evidence that E2 enhances myeloid DC development and maturation which heightens autoimmunity in animal models, providing one explanation for the increased incidence of MS seen in females post-puberty (see Figure 2).

### Neonatal mice are resistant to EAE compared to adults

It has been observed that mice of pre-pubertal ages are resistant to both adoptive transfer EAE and active EAE (79–82). For example, when the same number of lymph node cells are obtained from EAE donor mice and transferred into SJL/J or C57BL6/J recipient female mice of different ages, mice of pre-pubertal ages (1.5–4 weeks) develop a milder and delayed course of EAE compared to mice of post-pubertal ages (79, 80, 83). For studies in SJL/J mice, males showed a greater resistance to EAE than females after, but not before puberty, suggesting that female susceptibility to EAE is mediated in part by events that occur downstream of T cell priming (80). In adoptive transfer EAE studies in C57BL6/J mice, EAE susceptibility could be restored in neonatal mice by transferring in adult T cell-depleted splenocytes, but not purified B cells (79, 83), suggesting that post-pubertal myeloid cells or DCs were required to reactivate myelin-specific T cells to mediate CNS lesion formation. These studies also reported that MHC II expression in the CNS and spleen compartment increases in the female mice starting at approximately five weeks of age, corresponding to the age of pubertal onset.

Pre-pubertal SJL mice are also resistant to active EAE induced by immunization with PLP p139–151 peptide and CFA compared to older post-pubertal mice and this resistance is apparent until five weeks of age (79, 82, 83). In a study by Hoffstetter and colleagues using the PLP p139–151-induced model of EAE in female SJL mice (82), it was found that while two-week-old SJL mice could mount a myelin-specific Th1 response, Th17 responses were severely compromised, correlating with a reduced ability of neonatal macrophages to produce IL-12p40 (82). Therefore, higher IL-12p40 (or IL-12p70/IL-23) production by myeloid cells is a likely contributor to the enhanced myelin-specific Th1/Th17 immunity seen in SJL mice post-puberty (see Figure 2).

Active EAE development has also been compared between neonatal and adult mice of the C57BL6/J strain. In this model, EAE is induced by immunization with myelin oligodendrocyte glycoprotein peptide encoding amino acids 35–55 (MOG p35–55) and CFA (79, 83). Two-week old female mice are more resistant to EAE than adults, correlating with a reduced expansion of MOG p35–55-specific Th1 and Th17 cells in the periphery of these mice (79, 83). The defect in Th1/Th17 expansion did not relate to intrinsic defects in the T cells; In fact, the two-week-old T cells appeared to be hyper-responsive compared to the adult T cells (79). Instead, similar to the findings for pre-pubertal oophorectomy (68), DC2 cells were found to be under-represented in the spleens of two-week-old mice (79). Furthermore, DC2 cells from post-pubertal-aged adult mice exhibited higher MHC II and CD40 expression compared to pre-pubertal mice (79). Higher MHC II expression was also seen in B cells in the post-pubertal- compared to pre-pubertal-aged mice (79). Further, when neonatal splenocytes were stimulated with LPS, the neonatal cells produced lower levels of pro-inflammatory cytokines, including IL-12p40, and higher IL-10 than adult cells, suggesting a skewing of the post-pubertal cells towards a less immunogenic and more anti-inflammatory phenotype.

To further delineate whether these differences in the APC compartment were driving reduced Th1/Th17 responses in the neonates, Hertzenberg et al. (79) co-cultured T cell-depleted splenocytes (mainly B cells) as APCs with MOGp35–55-specific TCR transgenic cells, sourcing splenocytes or T cells from two-week old or adult mice. They found that APCs from young mice were inefficient at promoting Th17 proliferation and cytokine production by the responding MOG p35–55-specific T cells, whereas Th1 responses were relatively intact. Interestingly, the young APCs could uniquely promote the development of anti-inflammatory Th2 cells and Treg when the responding T cells were also from young mice. This suggests that the anti-inflammatory properties of APCs coupled with the intrinsic characteristics of CD4^+^ T cells were contributing to resistance to autoimmunity in the two-week old mouse (see Sections “Pre-pubertal CD4^+^ T cells exhibit a higher reactivity to self-antigens that is balanced by increased Treg activity prior to puberty” and “Th2 responses dominate in neonates and gradually shift towards Th1 with age, with Th1 responses becoming more dominant in females post-puberty” for further discussion about how Th2 and Treg responses are enhanced in neonates).

Altogether, these findings in neonates phenocopy the observations in pre-pubertal oophorectomized mice and suggest that puberty may enhance EAE susceptibility by increasing the maturation of DC2 cells and B cells. This maturational shift gives post-pubertal APCs the cellular machinery to break T cell tolerance more easily in response to infection. A drawback of these studies is that the immune phenotype was only studied in female mice and the effect of puberty was not specifically examined.

### Mice that are transgenic for myelin specific TCRs are vulnerable to EAE in the weeks immediately following onset of puberty

A number of lines of transgenic mice have been developed that express myelin-specific TCRs [reviewed in Ref. (61)]. In these mice, the large majority of the CD4^+^ T cell pool (upwards of 85%–90%) express a TCR that has a specific reactivity against a myelin antigen in the context of host MHC II. Each of these TCR transgenic lines can be used to study how one myelin-specific T helper cell clone can develop, be activated, or alternatively become tolerant within the host organism. Myelin-specific TCR transgenic mice bred on the C57BL6/J background exhibit a low incidence of EAE when housed under specific pathogen-free conditions [e.g., Refs. (84, 85)]. When EAE develops in these strains, it is due to cross-activation of the T cells by microbes present at mucosal sites (86, 87).

Of relevance to puberty, if myelin-specific TCR transgenic mice develop EAE, it occurs between five-to-ten weeks of age (87, 88). For example, in 2D2 MOG-specific TCR transgenic mice, EAE incidence peaks between five-to-seven weeks of age, with a slightly higher incidence of disease in the females (88) (Figures 1D,E). A similar age of onset of EAE has been reported for TCR transgenic mice that express the human HLA allele and a TCR specific for myelin basic protein (MBP) (87). A deeper exploration of the immune compartment in the latter mice revealed that post-puberty, MBP-specific TCR transgenic T cells had activities that were more skewed towards an effector phenotype. Isolated spleen cells from five-to-ten-week-old MBP TCR transgenic mice exhibited higher antigen-specific proliferation and IFN-γ, IL-17, and GM-CSF production *in vitro* compared to spleen cells of pre-pubertal counterparts. Five- to ten-week-old TCR transgenic mice also exhibited a reduced frequency of Treg in the spleen compared to pre-pubertal aged mice. The underlying reasons for the shift from Treg to T effector status with puberty is unclear but could relate to a decline in the thymic output of MBP TCR transgenic Treg or to the homeostatic proliferation of the MBP TCR transgenic cells that may occur post-puberty with thymic involution (see Figure 2 and Section “Puberty induces thymic involution and may shift the delicate balance between tolerance and autoimmunity” for a further discussion). Alternatively, it could relate to the developmental pattern of MBP expression, which increases in the murine thymus starting at four weeks of age; this could conceivably support increased positive selection of MBP TCR transgenic CD4^+^ T cells (90). Interestingly, beyond ten weeks, myelin specific TCR transgenic mice on the C57BL6/J background become resistant to EAE due to the T cells becoming anergic (87, 88). In conclusion, studies of myelin specific TCR transgenic mice have provided important insights into how the T cell compartment develops and how tolerance may be broken after puberty.

## How else does the immune system change with puberty or from childhood to adolescence?

It has been appreciated for some time that neonates have a more limited ability to develop T and B cell responses compared to adults in response to vaccination or pathogen challenge (91–93). To understand this phenomenon, a number of studies have contrasted the immune system in humans and mice of different ages (including prior to and after puberty). These studies revealed that immune system matures in some respects before puberty, but that this maturation continues through the peri-pubertal period. In this section, we will overview these studies according to immune cell process, while simultaneously highlighting how gonadal hormones impact these immune cells to convey sex differences in adaptive immunity post-puberty.

### The potential of pDCs to make IFN-α is increased post-puberty and regulated by E2

pDCs are a sub-population of DCs that are highly specialized for production of type I IFN. Increased IFN-α signaling by pDCs is a hallmark of systemic lupus erythematous (94) and signaling *via* the type I IFN-receptor is dysregulated in immune cells of MS patients (95). IFN-α has well known effects in promoting Th1 immunity by a variety of mechanisms during viral infection [reviewed in Ref. (96)]. It has been reported that pDCs from pre-pubertal female humans have higher potential to make IFN-α compared to pre-pubertal males in response to toll-like receptor (TLR7)/TLR8 stimulation (97). The potential of pDCs to produce IFN-α increases further in females after puberty, correlating with higher TLR7 expression by these cells (97). This sex difference in IFN-α production by pDCs prior to puberty may relate to X chromosome gene dosage (97), whereas the increase seen in females with puberty is likely due to estrogen actions on these cells. Studies by Seillet and colleagues showed that estrogen replacement in post-menopausal women enhanced IFN-α production by pDCs in response to TLR7 or TLR9 stimulation (74). Furthermore, mice that are deficient in ERα in the DC compartment, but have intact ovaries, have blunted pDC IFN-α production in response to TLR7 or TLR9 ligands (74). These *in vivo* effects of E2 are not recapitulated by transient *in vitro* treatment of pDCs with E2, which has led to the speculation that E2 changes the epigenome in pDCs in the bone marrow, which then specifies the descendent pDCs to be better IFN-α producers (74). Therefore, higher IFN-α levels post-puberty could be another contributing factor to the heightened myelin-specific Th1 immunity seen post-puberty (see Figure 2).

### Pre-pubertal CD4^+^ T cells exhibit a higher reactivity to self-antigens that is balanced by increased Treg activity prior to puberty

A number of studies have contrasted the phenotype of neonatal vs. adult CD4^+^ T cells. In earlier studies, it was concluded that neonatal T cells are hypo-responsive. This notion was born out of observations that neonatal lymph node cells or total T cells proliferated less and produced lower levels of cytokines when activated *in vitro*, as compared with adult T cells upon stimulation (98). However, this difference in neonate and adult T cell responses is largely due to the adults having a higher proportion of memory T cells in the T cell pool. When recent thymic emigrant (RTE) naïve CD4^+^ T cells are isolated from neonatal or adult mice and characterized, neonatal and weaning-aged CD4^+^ T RTEs have a strikingly higher TCR reactivity to self-antigens compared to post-pubertal CD4^+^ T RTEs as evidenced by higher expression of CD5 and Nur77 (99). This suggests that the TCR responsiveness of CD4^+^ T cells goes down with puberty. It is speculated that hyper-responsiveness to TCR signals and IL-7 (100) is what enables the RTE CD4^+^ T cells to expand rapidly in the lymphogenic environment to quickly fill the empty T cell niches at a time when the newborn is being exposed to many new antigens in its environment (99, 101, 102).

If RTE CD4^+^ T cells in the neonate or perinate are hyper-responsive, why are young mice or children resistant to autoimmunity? This is likely because neonatal RTE CD4^+^ T cells are kept in check by neonatal Tregs which are not only more abundant, but also more suppressive compared to adult Tregs (98, 99). Neonatal murine Tregs also express higher levels of CD5, indicating that they also have a higher TCR reactivity to self-antigens (103). In experiments with mice, it was found that transferring adult-derived Tregs into newborn autoimmune prone (autoimmune regulator; AIRE^−/−^) mice had no impact in slowing autoimmunity, whereas transferring perinatally-generated Treg prevented the development of disease (104). In pediatric humans, Tregs also express higher levels of Foxp3 than adult Tregs (98). In experiments that cultured neonatal and adult human lymph nodes in the presence of Treg depleting antibodies, it was observed that depletion of Tregs unleashed a much higher level of T cell proliferation in neonatal compared to the adult cultures (98), further suggesting that neonatal human Tregs are more suppressive than adult Tregs. Tregs have been shown to comprise a much higher proportion of the CD4^+^ T cell pool in the neonate as compared to adults (98) and are preferentially localized at mucosal sites where newborn naïve CD4^+^ T cells are first encountering environmental antigens (98). Taken together, these findings suggest that neonatal Tregs have a stronger reactivity to self-antigens that helps them reign in hyperactive non-Treg T cells to prevent autoimmunity. However, more studies are required to understand how puberty changes Treg characteristics.

### Puberty induces thymic involution and may shift the delicate balance between tolerance and autoimmunity

The thymus, the organ where new T cells are generated, reaches its maximal size just prior to puberty (105). At this time, the thymic output of new T cells and the frequencies of CD3^+^, CD4^+^ and CD8^+^ T cells in the blood are equivalent between males and females [reviewed in Refs. (11, 106)]. After puberty, the thymus undergoes a drastic decrease in size, called thymic involution, that is characterized by a rapid deterioration of thymic stroma, a decline in thymopoiesis, and reduction in the output of RTE CD4^+^ and CD8^+^ T cells that continues into old age [reviewed in Refs. (105–107)]. Studies in mice have shown that the involution occurs in response to suppressive effects of E2 and testosterone on thymic epithelium (108), but also because of suppressive effects of these hormones and LH on progenitors in the bone marrow [reviewed in Ref. (107)] (109). One possible consequence of this rapid decline in thymic output is that the existing T cell pool may undergo homeostatic proliferation in the lymphoid organs to compensate for the loss of incoming RTEs. Homeostatic regulation is associated with upregulation of memory markers on T cells and homeostatic proliferated cells respond more rapidly to antigen challenge due to a lower requirement for co-stimulation (110–112). In conjunction with the increased homeostatic proliferation, in the weeks following puberty, the frequency of thymic-derived Treg undergoes a decline that is disproportionately steeper than that of non-Treg CD4^+^ T cells (113), which may explain the decline of MBP-specific Tregs is seen in humanized HLA MBP TCR transgenic mice post-puberty (87). Thus, homeostatic proliferation of neonatal and perinatally-generated CD4^+^ T cells coupled with the steep decline in thymic Tregs could create a window of increased susceptibility to autoimmunity post-puberty.

There is also evidence that thymic involution post-puberty is steeper in males than in females. This results in females having an overall higher percentage of RTEs and a higher ratio of CD4^+^ to CD8^+^ T cells compared to males (105, 114–116). While the decline in thymopoesis is steeper in males, AIRE^+^ medullary thymic epithelial cells, which mediate the negative selection of T cells bearing a high affinity for self-antigen, undergo a proportionally steeper decline in females than males with progression to middle age (114). AIRE expression is also enhanced in the thymus by androgens and repressed by E2 (117, 118). These findings point to possible sex differences in the efficiency of negative selection of T cells post-puberty, which could be an additional factor accounting for the increase in CNS autoimmunity seen in females post-puberty.

### Th2 responses dominate in neonates and gradually shift towards Th1 with age, with Th1 responses becoming more dominant in females post-puberty

The balance between Th1 and Th2 immunity has been implicated to be important in the development of MS. Auto-reactive T cells from MS patients produce higher levels of IFN-γ and other pro-inflammatory cytokines (119), while production of the Th2 cytokine IL-4 is protective against the development of EAE (120). Though neonatal mice can mount Th1 responses when vaccinated with strong adjuvants such as in EAE, when vaccinated with weaker adjuvants or in the context of allogeneic responses, neonates are more prone develop Th2 immunity [reviewed in Refs. (91, 92)]. Thus, a proneness to develop Th2 responses could be an additional factor for why MS is rare prior to puberty.

Studies that examined the potential of human thymocytes or murine CD4^+^ T cells to produce cytokines in response to PHA at different ages concluded that the shift from Th2 towards Th1 is gradual and starts even prior to puberty (<five years of age in humans and less than one week in mice) (121). Part of the defective Th1 response in neonates relates to a reduced ability of Th1 cells to survive primary antigen challenge; while a mixed Th1/Th2 response is seen after primary immunization in neonatal mice, only Th2 cells are detected when the same mice are re-challenged with antigen (122). This occurs because of the increased susceptibility of neonatal Th1 cells to undergo IL-4 induced T cell apoptosis due to their unique expression of a heterodimer of IL-13Rα1 and IL-4Rα (122). With maturation, the increased IL-12 production by DCs inhibits IL-13Rα1 expression on T cells, thus interfering with this mechanism. In addition, neonatal CD4^+^ T cells also are epigenetically more poised for Th2 differentiation having decreased methylation at *cis*-acting sites in the Th2 cytokine locus and increased methylation in the *Ifng* promoter region (122).

Whether puberty further shifts the potential of Th cells to make Th1 cytokines is not known. What is known is that female CD4^+^ T cells produce higher levels of Th1 cytokines than male cells post-puberty [reviewed in Ref. (123)]. The enhanced Th1 response in females is in part related to increased IL-12p40 production by female APCs post-puberty. Indeed, in the case of murine peritoneal macrophages, it was shown that the sex difference in IL-12p40 production could be negated by castrating the male mice (124). In addition to these effects on IL-12p40, there is evidence that female Th cells are more poised to secrete IFN-γ compared to male T cells post-puberty (125–127). This sex difference is due to direct effects of E2 on the *Ifng* promoter (128) and to effects of androgens in repressing *Ifng* expression through induction of peroxisome proliferator-activated receptor-α expression in male T cells (125, 129). Although naïve CD4^+^ T cells do not express high levels of the IL-12 receptor, the expression of this protein is upregulated upon T cell activation and is important for Th1 lineage specification. In this regard, androgens repress and estrogens enhance IL-12-STAT4 signaling in T cells (130, 131). In addition, male SJL mice are more prone to develop type 2 innate lymphocyte (ILC2) responses that promote protective Th2 responses in EAE (discussed below in Section “ILC2 cells decrease in the blood and sex differences in these cells emerge post-puberty due to androgen regulation”) (132).

Thus, there is evidence that neonatal mice and young children have a Th2 biased immune response that is protective against autoimmunity and that females exhibit an increase in the Th1-immune response after puberty.

### ILC2 cells decrease in the blood and sex differences in these cells emerge post-puberty due to androgen regulation

Innate lymphoid cells (ILCs) are innate cells that develop from common lymphoid progenitors in the bone marrow and lack rearranged antigen receptors. These cells interact with their environment through a number of activating, inhibitory, and cytokine receptors (133). These interactions serve to amplify adaptive immune responses through cytokine production and cytotoxic killing of stressed cells, including tumors and virally infected cells (133). There are five known ILCs populations: natural killer (NK) cells, type I, 2, and 3 ILCs, and lymphoid tissue inducer cells (133). Of these subsets, there is strong evidence that type 2 ILCs (ILC2s), which express high levels of Th2 cytokines IL-5 and IL-13, are exquisitely sensitive to androgens, may undergo changes in numbers and phenotype with puberty, and contribute to the sex balance in Th1/Th2 inflammation seen post-puberty.

ILC2s strongly express the AR, but not ER-α and ER-β (134). The frequency of these cells in the peripheral blood of humans undergoes a steep decline between pre- to post-pubertal ages (135). The decline in ILC2 levels with puberty is likely due to the suppressive effect of androgens on ILC2 development in the bone marrow (136, 137). There also exists a sex difference in ILC2 frequencies in the blood of humans with females having higher numbers compared to males after the age of 10 (135). In mice, ILC2s become more abundant in the lung and visceral adipose tissue post-puberty, particularly in the females (137). While part of the increase in ILC2s in the lung has been shown to be age-dependent (136), there is also a striking sex difference in the number and phenotype of ILC2 that manifests after puberty (136). Post-pubertal female ILC2 cells have a slightly higher potential than male ILC2s to produce type 2 cytokines (IL-5 and IL-13) in response to PMA/Ionomycin, in part related to a higher predominance of the killer-cell lectin like receptor (KLRG1) negative ILCs (136). KLRG1 may limit ILC2 responses through immunoreceptor tyrosine-based inhibitory motif signaling in response to interactions with E-cadherin on endothelial cells (136). In addition to having lower KLRG1, female ILC2s in the lung also exhibit higher expression of activation and proliferation markers, CD25 and Ki67, suggesting these cells proliferate more extensively in the female tissues. In contrast, male ILCs express higher levels of the IL-33 receptor and therefore may be more responsive to IL-33 (136), which is an alarmin that is released during tissue damage and activates ILCs.

While the higher numbers of ILC2s seen in lungs of post-pubertal females is thought to be driving the female prevalence of type 2 allergic inflammation in this organ post-puberty (137), a higher abundance of ILC2 cells in the draining lymph nodes of males is thought to protect this sex from development of EAE through promotion of Th2 responses (132, 138). Androgens also stimulate ILC2 activity through induction of IL-33 production (132). Given the large influence of ILC2s on Th2 immunity, the sex difference in the abundance and maturation of these cells at mucosal sites and their responsiveness to IL-33 makes these cells candidate regulators of the sex difference in CNS autoimmunity seen post-puberty (see Figure 2).

In contrast to these findings for ILC2 cells, there is no strong evidence that NK cell numbers change with puberty (139, 140). Though these cells express high levels of ER-α, it is controversial whether E2 enhances or inhibits their activity (140). Furthermore, the frequencies of ILC1 and ILC3 cells do not change drastically in the blood of children from pre- to post-pubertal ages (135) and these cells express only low levels of hormone receptors (140).

### Neonatal CD8^+^ T cells exhibit higher TCR responsiveness and preferentially mature into short-term effectors instead of long-lived memory CD8^+^ T cells.

Though CD4^+^ T cells have been the focus of most MS immunological studies, pathological studies in MS have revealed that CD8^+^ T cells outnumber CD4^+^ T cells in the brain white matter (141). These cells appear to have a tissue resident memory phenotype (141), are clonally expanded (142), and persist at perivascular cuffs at the rim of the chronic-active lesions juxtaposed to activated microglia (141). It is speculated that the CNS residency of memory CD8^+^ T cells and lingering microglia activation may spur neuronal damage and disease progression in MS (141).

In this regard, adult naïve CD8^+^ T cells are better at forming CD8^+^ T cell memory cells compared to neonatal counterparts [reviewed in (143)]. In a study that co-transferred neonatal and adult CD8^+^ T cells into the same recipient mice, it was found that upon pathogen challenge neonatal CD8^+^ T cells were skewed towards a short-lived effector fate, whereas adult T cells differentiated into both effector and memory CD8^+^ T cell subsets (144). Upon pathogen re-challenge, the immune response was dominated by the adult CD8^+^ T cells (145). This lower potential of neonatal CD8^+^ T cells to form long-lived memory T cells relates to their higher TCR responsiveness, which is set by the differential expression of a number of microRNAs that regulate genes involved in TCR signaling, T cell proliferation, and cytokine production [reviewed in Ref. (143)]. In contrast to the situation of CD4^+^ T cells, where the TCR responsiveness declines between pre- and post-pubertal ages, CD8^+^ T cell TCR responsiveness declines gradually throughout the perinatal and pubertal period suggesting it is more of an age-dependent process (99). Nonetheless, the reduced ability of autoreactive CD8^+^ T cells to develop into long-lived memory cells prior to puberty could be an additional reason why young children are more resistant to MS (see Figure 2).

### Pre-pubertal mice develop less efficient humoral immune responses due to deficiencies in T cell help and intrinsic differences in the B cell compartment

B cell depletion with CD20 antibodies is a highly effective therapy in MS. It isn't entirely clear how these therapies inhibit MS disease activity, but the B cell pool that returns post-depletion is known to have a less mature phenotype with an increase in the frequency of naïve and transitional B cells and a decrease in memory B cells (146). The B cell pool in neonates resembles this state, as it is comprised mainly of naïve B cells and transitional B cells (147, 148). This difference in the proportion of B cell subsets between the neonate and the adult is speculated to be the reason why the neonatal B cells are also less efficient at presenting antigens to T helper cells (147, 148). Although a number of studies have reported finding higher levels of co-stimulatory molecule expression on B cells in adults vs. neonates, this is not the case when expressions on individual B cell subsets are directly compared between these groups (147, 148).

There is also evidence that humoral immune responses are compromised in neonates compared to adults [reviewed in Ref. (149)]. Vaccination of young children with T cell-dependent antigens results in only short-lived protection from pathogens, with reduced IgG titers that quickly wane and B cells that fail to form B cell memory (150). This is in part due to reduced T cell help provided by T follicular helper (Tfh) cells (151), a CD4^+^ T cell subset that interacts with B cells in secondary lymphoid organs and is crucial for promoting germinal center formation. When one-to-three-week-old mice are immunized with a T cell-dependent antigen, Tfh cells increase in numbers, but the magnitude of this response is severely compromised. Tfh cells fail to appropriately migrate into germinal centers (GCs), which appears to be due to intrinsic defects in the young Tfh cells (151). A comparison of Tfh cells, and GC and plasma B cells in immunized one week- (neonates), three week- (weaning age, pre-pubertal), and six-to-eight week- (post-pubertal) old mice showed that while the number of Tfh and GC cells generated by immunization increase between one- and three-weeks of ages, plasma cell numbers and Tfh percentages jumped from weaning age to the young adult (151), strongly implicating a role for puberty in Tfh development and the maturation of the GC B cell response (see Figure 2).

There is also evidence that in addition to Tfh cells, intrinsic differences in the B cells contribute to defective humoral immunity in neonates. Neonatal B cells like their T cell counterparts are hyper-responsive to BCR stimulation due to enhanced proximal BCR signaling (147). This may be due in part to the higher density of surface IgM (149), but also to a higher propensity of activated neonatal B cells to downregulate CD22; which is a negative modulator of BCR signaling (152). As a result of this altered BCR signaling, neonatal B cells proliferate more rapidly than adult B cells, but are also more prone to undergoing apoptosis and are inefficient at forming B cell memory (147, 148, 152). Neonatal B cells though able to produce IgM and IgA, are less efficient at antibody class-switching to IgG (147, 148) (Figure 2), thereby also explaining why pre-pubertal mice develop defective myelin-specific IgG responses during EAE (36).

Similar to the situation for the Tfh cells, the shift from a neonatal to adult B cell phenotype is in part age-dependent, gradually increasing from the neonate to four weeks of age (153). Though there is a paucity of studies that examined the effect of puberty on B cells, there is strong evidence that humoral immunity increases after puberty, particularly in females. Females exhibit higher IgG antibody levels in the steady state and upon infection or vaccination exhibit higher IgG titers and numbers of memory and plasma B cells than males (11, 154, 155). Though this sex difference is in part likely due to effects of E2 on T cell help (156), seminal studies done in a murine DNA-specific BCR transgenic model have demonstrated that E2 has direct effects in breaking B cell tolerance [reviewed in Ref. (157)]. These BCR transgenic mice have an abundance of B cells that recognize double-stranded DNA, but do not cause autoimmunity because of efficient negative selection of B cells that have the highest affinity for antigen (157). E2 treatment promotes the survival of these high-affinity B cells resulting in increased anti-DNA IgG levels in the blood and the development of lupus-like pathology in the kidneys of these mice (158). E2 mediated this effect by promoting the expression of molecules (Shp1 and CD22) that increase the threshold required for antigen-mediated B cell deletion (159). In addition to regulating the threshold of B cell activation, E2 also directly activates the transcription of activation-induced cytosine deaminase, an enzyme that is critical in regulating class-switch recombination and somatic hypermutation in B cells (160). Thus, the rise in E2 that occurs with puberty may hasten MS autoimmune mechanisms by not only enhancing the antigen presenting capacity of B cells, but also by promoting antibody-dependent mechanisms of tissue damage, a process that occurs in about half of MS lesions (161).

## Conclusion

In conclusion, puberty serves as a point of inflection in autoimmune susceptibility in MS and EAE, particularly in females. There is evidence that disease activity in MS may increase in the peri-pubertal period. The increase in CNS autoimmunity relates to a plethora of immune changes that occur with the transition from childhood to adolescence including an increase in antigen presentation and cytokine production by DC and pDCs, increased Th1, Th17, and Tfh responses, and a higher propensity of B cells and CD8^+^ T cells to form long-lived memory (summarized in Figure 2). There is strong evidence that the effect of puberty on DC2 cells, pDCs, and B cells in females is mediated by E2, whereas both androgens and estrogens regulate the sex difference in the Th1 immune response seen post-puberty. There is also a paucity of studies that have directly compared the phenotype of immune cells in both males and females before and after puberty. Furthermore, the role of gonadotropins in regulating pubertal changes in the immune system is underappreciated and needs to be further studied.
